# Consequences of the genetic threshold model for observing partial migration under climate change scenarios

**DOI:** 10.1002/ece3.3357

**Published:** 2017-09-08

**Authors:** Marleen M. P. Cobben, Arie J. van Noordwijk

**Affiliations:** ^1^ Department of Animal Ecology Netherlands Institute of Ecology (NIOO‐KNAW) Wageningen The Netherlands

**Keywords:** evolution, genetic diversity, individual‐based model, passerines, selection landscape, spatially explicit

## Abstract

Migration is a widespread phenomenon across the animal kingdom as a response to seasonality in environmental conditions. Partially migratory populations are populations that consist of both migratory and residential individuals. Such populations are very common, yet their stability has long been debated. The inheritance of migratory activity is currently best described by the threshold model of quantitative genetics. The inclusion of such a genetic threshold model for migratory behavior leads to a stable zone in time and space of partially migratory populations under a wide range of demographic parameter values, when assuming stable environmental conditions and unlimited genetic diversity. Migratory species are expected to be particularly sensitive to global warming, as arrival at the breeding grounds might be increasingly mistimed as a result of the uncoupling of long‐used cues and actual environmental conditions, with decreasing reproduction as a consequence. Here, we investigate the consequences for migratory behavior and the stability of partially migratory populations under five climate change scenarios and the assumption of a genetic threshold value for migratory behavior in an individual‐based model. The results show a spatially and temporally stable zone of partially migratory populations after different lengths of time in all scenarios. In the scenarios in which the species expands its range from a particular set of starting populations, the genetic diversity and location at initialization determine the species’ colonization speed across the zone of partial migration and therefore across the entire landscape. Abruptly changing environmental conditions after model initialization never caused a qualitative change in phenotype distributions, or complete extinction. This suggests that climate change‐induced shifts in species’ ranges as well as changes in survival probabilities and reproductive success can be met with flexibility in migratory behavior at the species level, which will reduce the risk of extinction.

## 1 INTRODUCTION

Migration is a widespread phenomenon across the animal kingdom as a response to seasonality in environmental conditions, with a wide variety in migratory strategies across and within species (Dingle & Drake, 2007). In quite some species of birds, the young do not migrate in groups or families, clearly demonstrating that migratory behavior in space and time is endogenous (Liedvogel, Åkesson, & Bensch, 2011). Many of these migratory species actually show a geographical cline in migratoriness, meaning that the species’ area of distribution ranges from populations with migratory individuals to populations with residential individuals, following an environmental gradient (Newton, 2008; Sahashi & Morita, 2013). Partially migratory populations, in which both phenotypes co‐exist (Chapman, Brönmark, Nilsson, & Hansson, 2011b), can in such species occur between these two kinds of populations. The stability of partially migratory populations has long been debated (Chapman, Brönmark, Nilsson, & Hansson, 2011a). Clearly, under such conditions, all individuals carry the genetic migration program, yet it is differentially expressed between the migratory and residential phenotypes (Franchini et al., 2017). As reasons for the differential expression of the genes related to migratory behavior, most empirical studies point toward fluctuating differences in survival or fecundity between phenotypes (Rolandsen et al., 2017) and an important role for density dependence in the maintenance of the two phenotypes at the same location (Holt & Fryxell, 2011; Kaitala, Kaitala, & Lundberg, 1993; Kokko, 2011; Lundberg, 2013; Shaw & Levin, 2011).

In passerines, studies looking into the heritability of migratory behavior have concluded that the inheritance of migratory activity is currently best described by the threshold model of quantitative genetics (Pulido, 2007, 2011; Pulido & Berthold, 2003, 2010; Pulido, Berthold, & Van Noordwijk, 1996; Van Noordwijk et al., 2006). In a recent study by Cobben and Van Noordwijk (2016), a parsimonious model was used to investigate whether the inclusion of such a genetic threshold model for migratory behavior can explain the existence of partially migratory populations. They conclude that assuming a threshold model leads to a spatially and temporally stable zone of partially migratory populations under a wide range of demographic parameter values. While the specific location and width of this zone of partial migration vary with dispersal distances and the strength of density dependence, structural differences between phenotypes and density dependence are no prerequisites for obtaining such a zone. Cobben and Van Noordwijk (2016) investigate the equilibrium selection pressures on the threshold value across the species’ range under model initializations with the number of individuals and the level of genetic diversity at carrying capacity.

Global warming has profound effects on geographical species distributions, showing range shifts for many taxa as a result of changing environmental conditions (Chen, Hill, Ohlemueller, Roy, & Thomas, 2011; Parmesan & Yohe, 2003). Particularly, for migratory species, it is expected that arrival at the breeding grounds might be increasingly mistimed under global warming as a result of the uncoupling of long‐used cues and actual environmental conditions, with decreasing reproduction as a consequence (Both & Visser, 2001; Lameris et al., 2017; Miller‐Rushing, Høye, Inouye, & Post, 2010; Møller, Rubolini, & Lehikoinen, 2008). In this study, we investigate the consequences for migratory behavior and the stability of the zone of partial migration under the assumption of a threshold model for migratory behavior, under a wide range of climate change‐related scenarios. For this, we use the model of Cobben and Van Noordwijk (2016) with the purpose to provide testable hypotheses for empirically investigating the support for this threshold model in the field. In addition, we relax their initialization assumptions regarding the level of genetic diversity to investigate the consequences of a reduced availability of genetic variation for the model outcomes. Specifically, we look into these five scenarios: (i) initialization with zero genetic diversity, both with a universally beneficial threshold value and a locally beneficial threshold value; (ii) initialization of the species at only the extreme ends of the landscape, so with migrant populations or resident populations only, to investigate the speed of range expansion after initialization; (iii) improving winter survival probability from the viewpoint that an increased winter temperature will relax stressful environmental conditions; (iv) deteriorating winter survival probability from the viewpoint that species interactions can mismatch and predictability of the conditions is lowered; (v) deteriorating reproductive success for migratory individuals from the viewpoint that these will suffer most from unpredictability of the environment, resulting in mistimed arrival at the breeding grounds. We are interested in the relationship between the level of genetic diversity and flexibility of migratory behavior at the species level, and specifically explore the speed of changes in the frequency of migratory phenotypes across generations following changes in environmental conditions, and the speed of colonization across the partial migration zone under initializations with different levels of genetic variation.

## 2 METHODS

We use the same model as in Cobben and Van Noordwijk (2016), a spatially explicit individual‐based metapopulation model of a haploid species with discrete generations, and the rules for determining the population dynamics are inspired by passerine ecology. The species has a single trait that determines the threshold for migration, which is allowed to evolve.

### 2.1 Landscape

The simulated landscape consists of 100 columns (*x*‐dimension) of 25 breeding patches each (*y*‐dimension), all with carrying capacity *K*. We assume bouncing borders in both *x* and *y* directions. Hence, an individual cannot leave the landscape by dispersal, but migratory individuals are assumed to not spend their winters in the landscape that signifies only the species’ breeding area. There are two gradients in the *x*‐dimension, of increasing winter survival and decreasing reproduction, simulating average conditions on a trajectory from the pole to the equator. The experienced nominal average winter survival for residents is 0.01 times the *x*‐location, so changes linearly from 0.01 at *x *=* *1 to 1 at *x *=* *100 (Equation (1)). Reproduction is corrected for position along the *x*‐dimension through a reproduction factor that decreases linearly from 1 at *x *=* *1 to 0.8 at *x *=* *100 (Equation (2)), incorporating the process that these species prefer more polar breeding areas where a high‐quality food peak can sustain larger broods. Both winter survival and reproduction are reduced by density‐dependent factors (see below for further details).

### 2.2 Winter survival and population dynamics

Local populations are composed of haploid individuals, each of which is characterized by a single trait, its migration threshold. If the local expected winter survival is lower than the genetically determined threshold *T*, the individual migrates (Able & Belthoff, 1998). This expectation includes all processes determining winter survival, so also population density as explained below, and thus models the process that an individual makes an estimate, ahead of time, of whether local winter conditions will be good enough to reach its own endogenous threshold in survival probability, based on environmental cues. A migrant is subjected to a constant probability of winter (thus migration) survival *s*_*m*_. In contrast, a resident can survive the winter locally with probability *s*_*r*_ that is determined by its *x*‐location. Resident winter survival is in addition controlled by the local resident density, so in all determined according to the following equation:

(1) $$s_{r}=0.01x\left(1-\left(\left(1-c\_dens_{s}\right)\frac{Nr_{x,y,t}}{K}\right)\right)$$

where *x* is the *x*‐coordinate of the patch [1,100]; *Nr*_*x,y,t*_ is the number of resident individuals in patch *x, y* at time *t*;* K* is the carrying capacity; *c_dens*_*s*_ governs the strength of density dependence, with no density dependence at *c_dens*_*s*_ = 1, and *s*_*r*_ = 0 under *c_dens*_*s*_ = 0.6 and density = 2.5 (Fig. S1a in Appendix A and Table 1). In our simulations, the winter survival of migrants *s*_*m*_ was 0.5. In our model, the migrant survival is not determined by local winter population densities, as individuals are known to move further south if the local carrying capacity is reached. In addition, migrant survival is thought to be mostly determined by mortality during migration.

**Table 1:** Parameter values

Parameter/variable	Value	Meaning
Individual variables:		
*T*	Evolving	Migration threshold
Simulation parameters:		
*K*	100	Carrying capacity
*s*_*m*_	0.5	Migrant survival
*c_dens*_*s*_	0.6	Strength of survival density dependence
*c_dens*_*r*_	0.4	Strength of reproduction density dependence
*c_loc*_*r*_	0.8	Strength of reproduction location dependence
*m*	10^−4^	Mutation rate
*d*	0.1	Dispersal probability
δ	2	Maximum dispersal distance
*x*_*max*_	100	Extent of simulated landscape in x‐direction
*y*_*max*_	25	Extent of simulated landscape in y‐direction

If an individual survives the winter season, either as a resident or a migrant, it can disperse from its native patch with probability *d* to a neighboring cell with a maximum dispersal distance of δ cells. It then produces a number of offspring. This number is randomly drawn from a discrete uniform distribution with a minimum value of zero and a maximum of six, and then corrected for density and location, leading to the following equation:

(2) $$R=\text{No}\left(1-0.01x\left(1-c\_loc_{r}\right)\right)\left(1-\left(\left(1-c\_dens_{r}\right)\frac{N_{x,y,t}}{K}\right)\right)=\text{No}\times c_{tot}$$

where No is drawn from *U* {0,6}; *N*_*x,y,t*_ is the number of individuals in patch *x,y* at time *t* (i.e., including both the migrants and residents); *c_loc*_*r*_ governs the sensitivity of reproduction with location *x*, with no dependence for *c_loc*_*r*_ = 1, and maximum dependence of *c_loc*_*r*_ = 0; *c_dens*_*s*_ governs the strength of density dependence, with no dependence at *c_dens*_*s*_ = 1; *c*_*tot*_ is then the total reproduction correction factor (Fig. S1b in Appendix A). After reproduction, the individual dies.

Although carrying capacity *K* is equal for both Equations 1 and 2, the population sizes and thus the densities change over the seasons. In addition, the parameters governing the strength of density dependence are different for both equations (*c_dens*_*s*_ and *c_dens*_*r*_, Table 1), which results in different effects of density on resident winter survival and reproduction, respectively.

### 2.3 Genetics

The individuals have one genetic trait, the migration threshold, which is located at a single gene. The threshold value *T* is a continuous number in the range [0,1]. The individuals are haploid and reproduce asexually, thus the offspring inherit the threshold values from their mothers. To allow for some variation, we apply a probability of mutation *m*. When such a mutation occurs, the threshold value is changed into a new randomly chosen value [0,1].

### 2.4 Simulation experiments

With our simulation experiments, we want to test how sensitive the model outcomes in Cobben and Van Noordwijk (2016) are to different model starting conditions (initializations) and abrupt changes in environmental conditions during the model run, inspired by projected conditions under climate change. Table 1 summarizes all relevant model parameters, their meanings, and the standard values used for the simulations. We investigated different climate change scenarios, first by different model initializations, both regarding number and location of individuals, and the genetic threshold values *T*. The initialization with individuals at low *x*‐values simulates a range expansion across the landscape starting from migratory populations only, while initialization at high *x*‐values investigates an expanding range from residential populations. A lack of genetic diversity for threshold values *T* could potentially change the model outcome, so we contrasted situations where diversity at initialization was either maximum or zero, with zero genetic diversity divided into two sets: *T *=* *0.5 and *T* is fitted. Fitted *T* means *T *=* *0.1 for residents and *T *=* *0.9 for migrants. In these fitted *T* scenarios, the species’ range expanded from either side of the landscape, with the initial *T* being strongly adapted to the local conditions at the extreme ends of the landscape. In addition, we investigated several abruptly changing environmental conditions after model initialization, leading to either changing survival or reproduction probabilities. Such changes could cause disruptions in previous model outcome by selecting for a different equilibrium, which we tested by subjecting residential individuals to 20% increased survival, assuming warmer winters, or 20% decreased survival assuming changes in timing of peak food causing a phenotypic mismatch with timing of breeding. Migratory individuals experienced 20% decreased reproductive success, assuming mistiming of arrival at the breeding grounds after spring migration. Table 2 summarizes all the different scenario options. From these, we constructed a set of relevant combinations, resulting in 24 different scenarios (for a full scenario overview see Table S1). Changing survival and reproduction conditions were implemented 300 years after the start of the model run, when demographic equilibrium was reached. The only exceptions to this are the scenarios implemented with populations initialized across the landscape in combination with zero genetic diversity, in order to prevent genetic diversity from increasing before changing conditions were applied. For each scenario, we performed 100 replicate simulations.

**Table 2:** Scenario options. All different scenario possibilities are defined by combining the three columns, resulting theoretically in 3 × 3 × 4 scenarios, of which we selected 24 relevant combinations

	Initialization for..		Abrupt changes
	Individuals	Genetic diversity	
Different model options	Maximum	Maximum, random: 0 < *T *≤ 1	None
	Residents only	Zero: *T *=* *0.5	Resident survival + 0.2
	Migrants only	Zero but fitted to a specific location: *T *=* *0.1 for residents, *T *=* *0.9 for migrants	Resident survival −0.2 Migrant reproduction −0.2

### 2.5 Analysis

The individual phenotypes, that is, migrancy versus residency, were documented in time and space throughout the simulations and summed per *x*‐location. The migration threshold values were averaged per *x*‐location. We defined the polar border of the partial migration zone as the smallest *x*‐location where the total number of residents was at least 1% of the total number of individuals. The equatorial border is equivalently defined as the largest *x*‐location where at least 1% of the total number of individuals was a migrant. The difference between both borders is the width of the partial migration zone, while the location of the zone was defined by its middle *x*‐ location.

## 3 RESULTS

**Figure 1:**
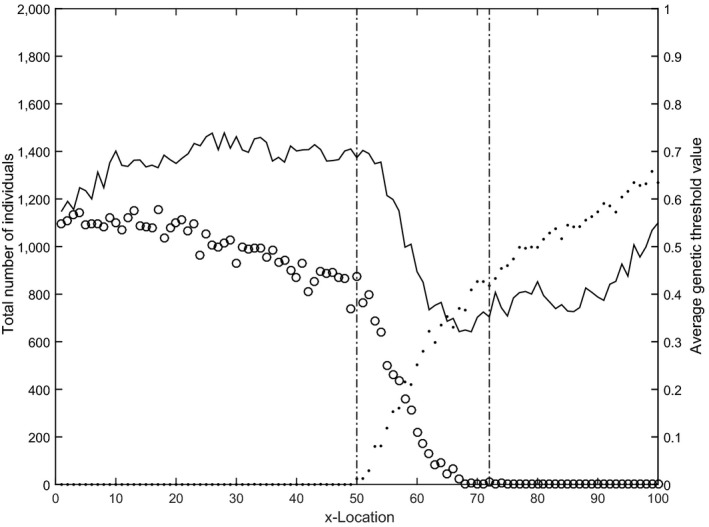
Typical example of population sizes at equilibrium. The number of individuals is summed per phenotype and over all 25 populations per x‐location. Open circles ○ indicate migratory individuals, dots • indicate residential individuals. The dotted lines indicate the borders of the zone with partially migratory populations. The solid black line shows the average threshold value *T* at equilibrium.

All runs of all scenarios converge to an equilibrium situation with fully migratory populations at the lower *x*‐values, fully residential populations at the higher *x*‐values, and a spatially and temporally stable zone of partially migratory populations between these (see Figure 1 for a typical example).

### 3.1 Range expansion scenarios

**Figure 2:**
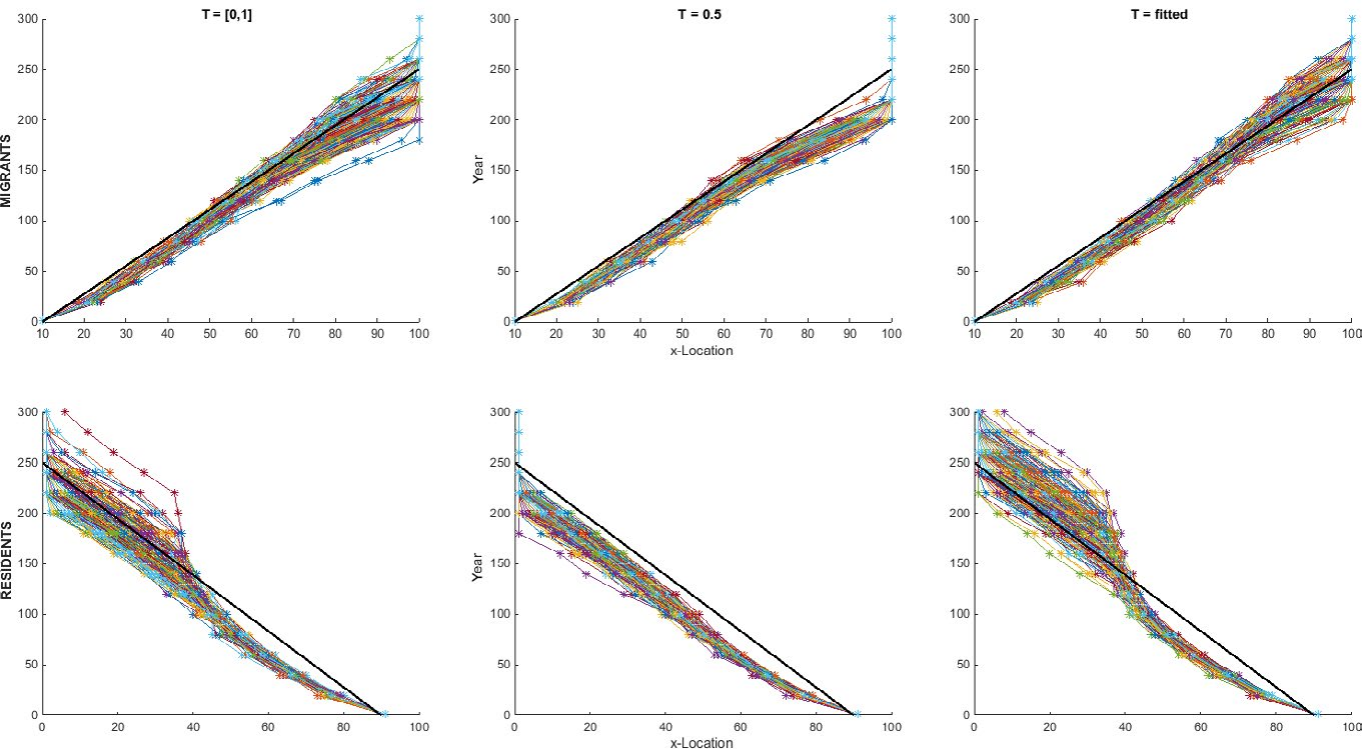
Range expansion scenarios. Each panel shows the location of the range front in time for all 300 runs of the different scenarios. The top row of panels is initiated with migrant populations (1 ≤ *x *≤* *10) and the bottom row with resident populations (90 ≤ *x *≤* *100). The panel columns each had a different level and value of genetic diversity at initialization, with *T *= fitted means *T *=* *0.9 for the migrant scenario, and *T *=* *0.1 for initialization with residents. The bold black line has the same slope in all panels to facilitate comparisons between scenarios showing, for example, that more model run lines in the *fitted T* scenarios end higher than this line than in the other scenarios, indicating a longer time period to full colonization

In the range expansion scenarios, the genetic diversity and location at initialization determine the species’ colonization speed across the zone of partial migration and therefore across the entire landscape (Figure 2). Expansion from an initialization with *T *=* *0.5 is fastest, followed by expansion after initialization with maximum genetic diversity, that is, *T * =  [0,1]. Average expansion speed is lowest after initialization with fitted *T*, which is *T *=* *0.1 for initialization with residents, and *T *=* *0.9 for migrants. *T *=* *0.5 results in a phenotype switch (the result of which can be seen in Figure 1) at *x *=* *50, which allows fast colonization of the zone of partial migration. After colonization, selection on the threshold value increases the number of migrants and thus moves the zone of partially migratory populations toward a larger *x*‐value, as increasing population densities benefit the migratory phenotype due to reduced average resident survival (Fig. S2). Initialization with full genetic diversity of *T * =  [0,1] results in an on average slower range expansion because colonization of the partial migration zone is limited by dispersal of individuals with a proper *T*‐value (i.e., coding for the beneficial phenotype at that location). When the populations are initialized with a fitted *T*‐value (*T *=* *0.1 for residents and *T *=* *0.9 for migrants), the colonization of the partial migration zone depends on the de novo mutation of a proper *T*‐value, which causes an on average lower colonization rate of the landscape. At *x *>* *50, the survival probability is higher for residents than for migrants that always have a survival probability of 0.5. This results in a greater expansion speed across the landscape up to the partial migration zone in the scenarios initialized with resident populations, in contrast to initialization with migrants only, independent of the level and value of genetic variation.

### 3.2 Scenarios with changing environmental conditions

**Figure 3:**
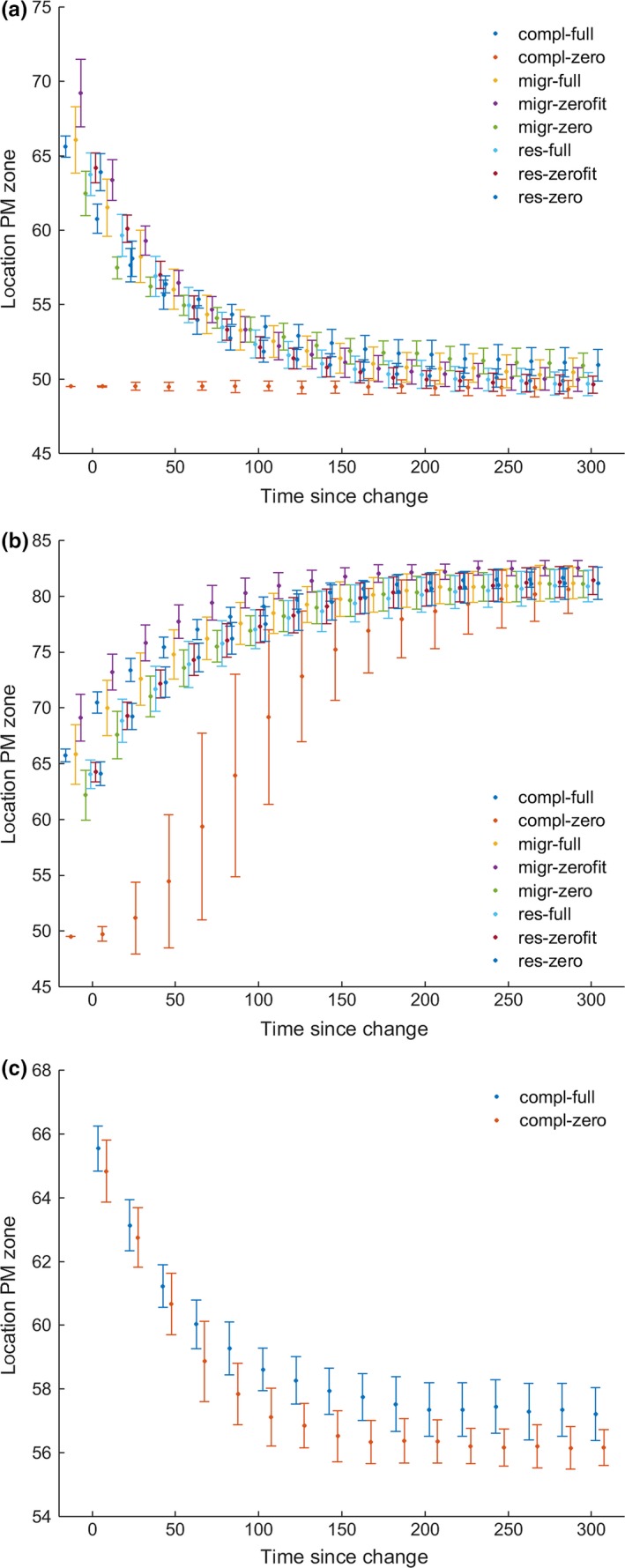
Scenarios with changing environmental conditions. The average *x*‐location of the zone of partial migration over 100 replicate runs, with the error bars expressing standard deviations, in time after the change in survival probability was abruptly implemented in the model simulation for eight different scenarios (a, b). Change was applied 300 generations after model implementation except for the “compl‐zero” scenario. Decreased reproduction for migrants (c) was simulated in two different scenarios after demographic equilibrium was reached at generation 300. “*Compl*” = initialized with individuals 1 ≤ *x *≤* *100, “*migr”* = 1 ≤ *x *≤* *10, “*res”* = 90 ≤ *x *≤* *100, “*full”* = initialized with 0 ≤ *T *≤* *1, “*zero”* = *T *=* *0.5, “*zerofit” = T *=* *0.9 (migrants) and *T *=* *0.1 (residents). a: resident winter survival increase of 20%. b: resident winter survival decreased of 20%. c: migrant reproductive success decrease of 20%

Abruptly changing environmental conditions after model initialization cause a change in the location of the zone of partially migratory populations (Figure 3) and in the total numbers of individuals, but never a qualitative change in phenotype distributions (i.e., the specific, or quantitative outcome could be different, but the general pattern is always the same) or the complete extinction of the passerine. The applied increase in resident winter survival of 20% causes a shift of the zone of partially migratory populations toward lower *x*‐locations as the residential phenotype has an increased benefit. As the resident winter survival is decreased by 20%, we observe the opposite effect with a shift of the PM zone toward higher *x*‐locations by an increase in the number of migrants as the migratory phenotype has increased benefit. A decrease in migrant reproductive success of 20% again causes a shift of the partial migration zone toward lower *x*‐locations with a larger number of fully residential populations due to increased fitness of this phenotype. Changing winter survival conditions in the scenario with individuals initialized in all populations with zero genetic diversity of *T *=* *0.5 (“*compl‐zero”*) was applied immediately after initialization to prevent genetic diversity from increasing during the model run and investigate the extreme circumstance of abrupt change without genetic diversity. Under winter survival increase (Figure 3a), the new equilibrium location of the zone of partial migration more or less equals the location after initialization, resulting in a stable location after environmental change. When winter survival decreases (Figure 3b), the location of the PM zone in the “*compl‐zero”* scenario needs to move furthest of all scenarios. This happens during a longer time period and with a larger standard deviation but eventually leads to the same result, without the occurrence of species’ extinction in any of the replicate runs. In this extreme scenario, we do see local population extinction (Fig. S3) as the combination of reproduction level and changed resident winter survival prevents the existence of stable resident populations near the zone of partial migration. These habitats of locally extinct populations of residents then need to become colonized by migratory individuals first. As the occurring *T *=* *0.5 codes for the migratory phenotype up to *x *=* *70 under the changed survival curve of the resident winter survival, the shift of the zone of partial migration is initially not limited by the de novo mutation of beneficial *T*‐values. However, as the population sizes increase again, the positive feedback loop between threshold values and number of individuals also occurs here, after which the individuals with higher *T*‐values (and thus migratory phenotype) can later replace residents at *x*‐values larger than 70 (Fig. S3).

## 4 DISCUSSION

Cobben and Van Noordwijk (2016) investigated the consequences of assuming a genetic threshold model of migratory behavior for the existence and stability of partially migratory populations. Here, we extend their study by relaxing their initialization conditions and investigating a range of climate change‐related scenarios to study the robustness of their findings. Our results show that assuming the genetic threshold model for migratory behavior allows rapid across generation changes in migration decisions at the landscape level and a spatially and temporally stable zone of partially migratory populations under all investigated scenarios. This suggests that if climate change‐induced shifts in species’ ranges as well as changes in survival probabilities and reproductive success are observed to be met with flexibility in migratory behavior at the species level, this indicates support for a genetic threshold model for migratory behavior and lowers the impact of these changes on population sizes. The mentioned flexibility should be interpreted as changes across generations in the decision whether or not to migrate in populations at specific locations in the landscape.

The effect of the level of genetic diversity in threshold values was dependent on the actual *T*‐values. When the threshold value in combination with the local survival conditions gave the most fit phenotype across most of the landscape (*T *=* *0.5), this would increase the expansion speed compared to higher levels of genetic diversity (0 ≤ *T *≤* *1, Figure 2). This was not the case when the threshold value was fitted to either migratory (*T *=* *0.9) or residential populations (*T *=* *0.1), in which scenarios the colonization depended on a de novo mutation of *T* and therefore took longer. Generally, the colonization of either empty habitat patches or patches inhabited by individuals of the wrong phenotype would be limited by the species’ dispersal capacity. Importantly, while populations were maladapted after the abrupt and extreme environmental changes, there was only one scenario *(“compl‐zero”)* in which this actually led to a temporary local population extinction (Fig. S3).

Rapid changes of migratory behavior in response to changing conditions are well documented. Many examples exist of changes in arrival and migration time, changes in migration routes and wintering areas, and also population shifts either toward more migratory or more sedentary and even residential behavior (Able & Belthoff, 1998; Berthold, Helbig, Mohr, & Querner, 1992; Fiedler, 2003; Knudsen et al., 2011; Partecke & Gwinner, 2007; Pulido & Berthold, 2010). This flexibility is often attributed to plasticity as opposed to evolutionary change in migratory behavior (Knudsen et al., 2011). However, the finding that the inheritance of migratory activity is best described by the threshold model of quantitative genetics (Pulido, 2011) reconciles these viewpoints regarding the issue of migratory versus residential behavior for species where migration is controlled endogenously. In this study, we investigate the evolutionary change in local threshold values that determine migratory behavior in combination with local survival conditions, which is thus a plastic trait. In migratory fish, this was studied by Sahashi and Morita (2013), who show that selection on the threshold value (in their case a threshold in size at maturity), resulted in a decreasing threshold value with increasing distance from the sea, leading to an increased number of residential individuals as migration costs increase. Also here, migratory behavior is plastic, as a fish of a certain size at maturity is migratory under downstream conditions, and residential in upstream populations, which is comparable to our trait, where a specific *T*‐value will give different migratory phenotypes under different environmental conditions.

Despite these observed rapid changes in migratory behavior in response to changing environmental conditions, it is widely acknowledged that migratory species in general seem to suffer more from population declines than species that do not migrate, with climate change as one of its four drivers (Wilcove & Wikelski, 2008). It is predicted that communities of migratory bird species in Europe will be altered through changes in migratory behavior under current projected climatic changes, rather than through reassembly of bird communities, the latter of which would lead to lower proportions of migratory species (Schaefer, Jetz, & Böhning‐Gaese, 2008). Interestingly, partially migratory populations have been observed to suffer less population declines than fully residential or fully migratory populations from changing climatic conditions (Gilroy, Gill, Butchart, Jones, & Franco, 2016), suggesting the migratory diversity facilitates adequate responses such changes. Our results suggest that for species of which migration decisions are controlled by the genetic threshold model, environmental changes could be met with across generational changes in the frequency of migratory phenotypes. Current findings that partially migratory populations seem to better deal with changing can be in line with our suggestion. We would then expect that fully residential and fully migratory populations are able to adapt as well. Current local lack of genetic diversity for different threshold values might delay the necessary changes and population declines could be the result ongoing selection against threshold values that are deleterious under the new conditions. Particularly in populations bordering the zone of partially migratory populations, selection against *T*‐values coding for the adverse migratory phenotype is strong (Cobben & Van Noordwijk, 2016). In this study, populations are well connected and the full environmental gradient is inhabited, allowing the build‐up and movement of genetic diversity for *T*‐values, which is not necessarily or even most likely not the case in the empirical examples investigated. All in all, from our study, we conclude that under the assumption of the genetic threshold value for migratory behavior, the phenomenon of (passerine) migration will not be lost provided species can inhabit places where resident survival is smaller than migratory survival, although reaching such a new equilibrium situation may take multiple generations.

We have used the trait of being migratory in a set of life history traits inspired by passerine ecology. We believe that the results are equally valid for other threshold characters in other life histories. The classical examples of threshold characters are insect traits, such as multivoltinism, diapause, and wing dimorphism (Matsumura, 1996; Saulich & Musolin, 1996; Söderlind & Nylin, 2011). Some of these traits, for example, diapause, also fulfill the condition that local densities for one phenotype are lower due to the existence and inherent absence of the other phenotype. Although density dependence is not essential for the maintenance of populations consisting of both phenotypes, it does substantially increase the area where mixed populations are observed (Cobben & Van Noordwijk, 2016). A major difference with the life history studied here is that in insects the reproduction is much higher. From this, one can expect an increase in the number of mutations which will likely cause an even wider zone of mixed populations. The modest maximum dispersal distance employed in this study is fairly agreeable to insect life history, in all implying that our model is also applicable to insect life histories and well‐known threshold traits in these.

Our model is artificial in a number of ways: We assume discrete generations and haploid inheritance. The results thus mainly provide a view of the selection landscape, in interaction with the limited mutation and dispersal rates. The result that shifts in the selection pressure lead to quick responses while maintaining stable mixed populations were obtained under a wide range of scenarios and parameter values. Another simplification of reality was made when assuming that individuals can perfectly predict what their local survival probability is, that is, they “know perfectly” where they are. This aspect has been thoroughly tested by Cobben and Van Noordwijk (2016), who show that decreased predictability of survival conditions has a small effect on the mean and standard deviation of the location of the partial migration zone, but does not cause a qualitatively different result.

In this study, we investigated the flexibility of migratory behavior at the landscape and species level, and stability of partially migratory populations under a set of different initialization conditions and climate change‐related scenarios. The results indicate that assuming a genetic threshold model for migratory behavior always leads to a spatial zone consisting of partially migratory populations and supports rapid changes of local observed migratory phenotypes, which is in agreement with empirical data.

## CONFLICT OF INTEREST

None declared.

## AUTHOR CONTRIBUTIONS

MC and AvN had the idea for the study, designed the model and the experiments. MC constructed the model and analyzed the data. MC and AvN interpreted the results. MC wrote the manuscript, taking AvN's comments into account.
