# Supplementary material for: Consequences of the genetic threshold model for observing partial migration under climate change scenarios
Source: Ecol Evol. 2017 Sep 8;7(20):8379–87. doi: 10.1002/ece3.3357 (PMC5648652; doi:10.1002/ece3.3357)

Appendix A: Additional figures

In this appendix we show some additional supporting figures and tables. In Figure S1 we show values of Equation 1 (S1a) and Equation 2 (S1b) as a function of *x*-location and for different values of the population density. Table S1 gives a full overview of all the scenarios and their features. Figure S2 shows a range expansion in time and space of a single run of the scenario initiated with individuals at 1 ≤ *x* ≤ 10 and *T* = 0.5, with a positive feedback loop between population size and *T*-value at the partial migration zone after it has been initially colonised by residential individuals. Figure S3 is a series of panels showing the time series of a single run of the scenario *‘compl_zero_decr’*. This is the only (and extreme) scenario in which there is temporarily local extinction after the winter survival probability is decreased by 20% immediately after initialisation with individuals 1 ≤ *x* ≤ 100 and zero genetic diversity with *T* = 0.5.

Figure S1a. The actual values of resident winter survival *s_r_* (Equation 1) as a function of
*x-*location and different values of $\frac{{Nr}_{x,y,t}}{K}$ , i.e. population density (dens), for parameter *c_dens_s_* = 0.6 (as used in the base scenario). In addition *s_r_* for *c_dens_s_* = 1, i.e. without density dependence.


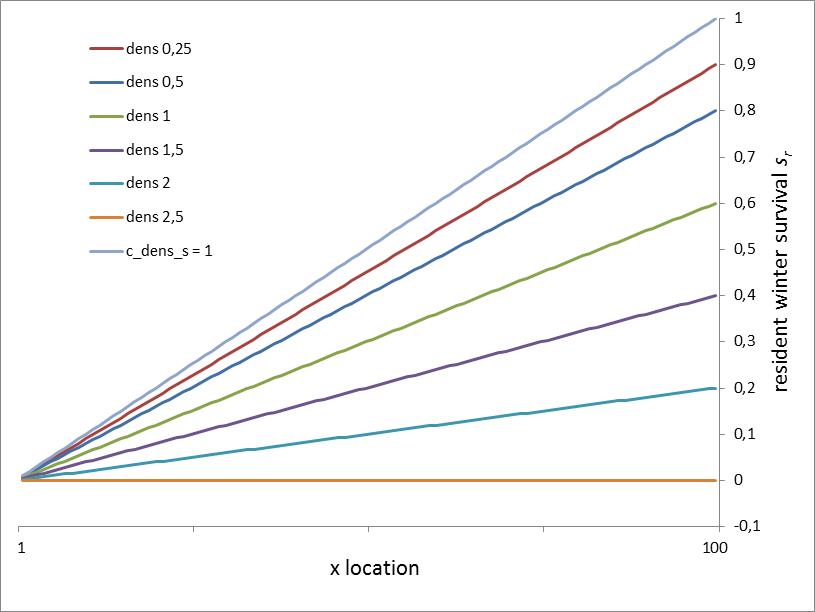


Figure S1b. The reproduction correction factor *c_tot_* (Equation 2) as a function of
*x-*location and different values of $\frac{{Nr}_{x,y,t}}{K}$ , i.e. population density (dens), for *c_dens_r_* = 0.4, and *c_loc_r_* = 0.8 (both as used in the base scenario).


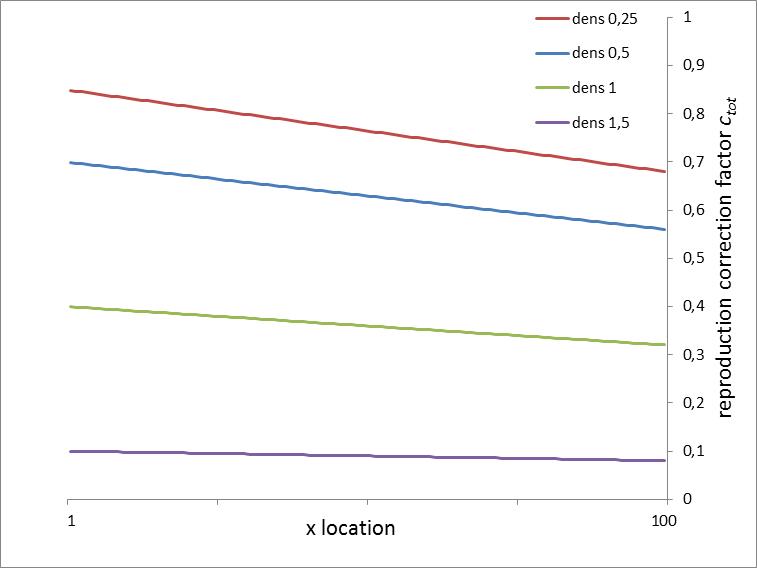


Table S1. Full scenario overview

| Name | Location initialisation | Genetic diversity initialisation | Change | Year of change |
| --- | --- | --- | --- | --- |
| compl_zero_incr | 1 ≤ *x* ≤ 100 | *T* = 0.5 | survival increase | 1 |
| compl_zero_decr | 1 ≤ *x* ≤ 100 | *T* = 0.5 | survival decrease | 1 |
| compl_zero_repr | 1 ≤ *x* ≤ 100 | *T* = 0.5 | reproduction decrease | 300 |
| compl_full_incr | 1 ≤ *x* ≤ 100 | 0 ≤ *T* ≤ 1 | survival increase | 300 |
| compl_full_decr | 1 ≤ *x* ≤ 100 | 0 ≤ *T* ≤ 1 | survival decrease | 300 |
| compl_full_repr | 1 ≤ *x* ≤ 100 | 0 ≤ *T* ≤ 1 | reproduction decrease | 300 |
| migr_zero_incr | 1 ≤ *x* ≤ 10 | *T* = 0.5 | survival increase | 300 |
| migr_zero_decr | 1 ≤ *x* ≤ 10 | *T* = 0.5 | survival decrease | 300 |
| migr_zero_none | 1 ≤ *x* ≤ 10 | *T* = 0.5 | none | *NA* |
| migr_full_incr | 1 ≤ *x* ≤ 10 | 0 ≤ *T* ≤ 1 | survival increase | 300 |
| migr_full_decr | 1 ≤ *x* ≤ 10 | 0 ≤ *T* ≤ 1 | survival decrease | 300 |
| migr_full_none | 1 ≤ *x* ≤ 10 | 0 ≤ *T* ≤ 1 | none | *NA* |
| res_zero_incr | 90 ≤ *x* ≤ 100 | *T* = 0.5 | survival increase | 300 |
| res_zero_decr | 90 ≤ *x* ≤ 100 | *T* = 0.5 | survival decrease | 300 |
| res_zero_none | 90 ≤ *x* ≤ 100 | *T* = 0.5 | none | *NA* |
| res_full_incr | 90 ≤ *x* ≤ 100 | 0 ≤ *T* ≤ 1 | survival increase | 300 |
| res_full_decr | 90 ≤ *x* ≤ 100 | 0 ≤ *T* ≤ 1 | survival decrease | 300 |
| res_full_none | 90 ≤ *x* ≤ 100 | 0 ≤ *T* ≤ 1 | none | *NA* |
| migr_zerofit_incr | 1 ≤ *x* ≤ 10 | *T* = 0.9 | survival increase | 300 |
| migr_zerofit_decr | 1 ≤ *x* ≤ 10 | *T* = 0.9 | survival decrease | 300 |
| migr_zerofit_none | 1 ≤ *x* ≤ 10 | *T* = 0.9 | none | *NA* |
| res_zerofit_incr | 90 ≤ *x* ≤ 100 | *T* = 0.1 | survival increase | 300 |
| res_zerofit_decr | 90 ≤ *x* ≤ 100 | *T* = 0.1 | survival decrease | 300 |
| res_zerofit_none | 90 ≤ *x* ≤ 100 | *T* = 0.1 | none | *NA* |

Figure S2a. The total number of individuals per *x*-location, expanding across the landscape from *x* = 10 for a single run of the scenario initiated with genetic threshold value *T* = 0.5. The coloured lines indicate time, with dark blue on the left is *t* = 1, one time step further orange line *t* = 20, and from there increasing with 20 generations per line up till *t* = 300. As such we can follow the range expanding border in time across the landscape.


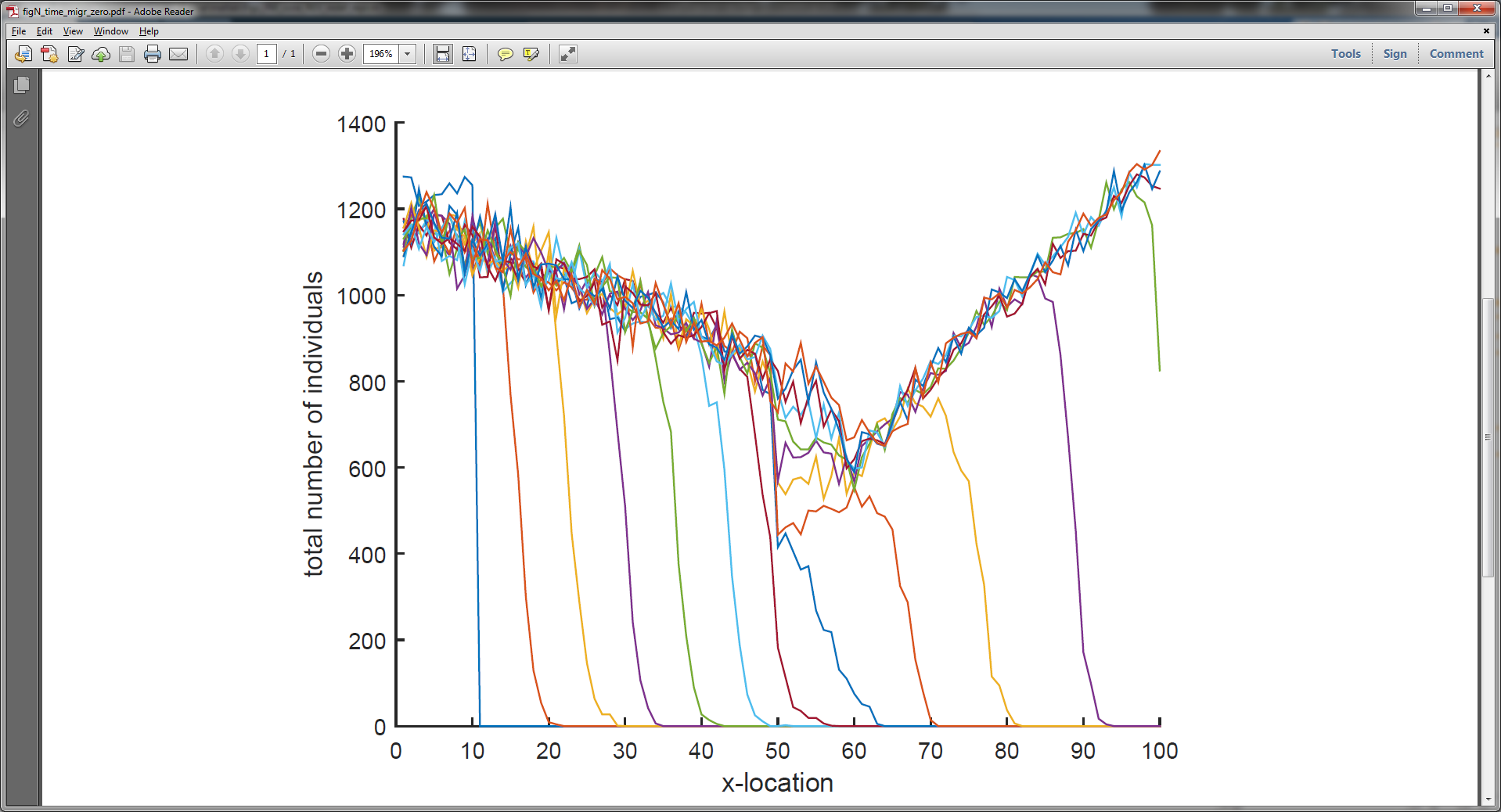


Figure S2b. The average genetic threshold value *T* per *x*-location for the same single run (see Figure S2a) of the scenario initiated with genetic threshold value *T* = 0.5. To facilitate visual comparison with Figure S2a we allow the threshold value to drop at the expanding range border. The coloured lines indicate the same time steps as Figure S2a. Here we can see that only after colonisation of the zone of partial migration, the average threshold value increases in a positive feedback loop with the number of individuals (Figure S2a).


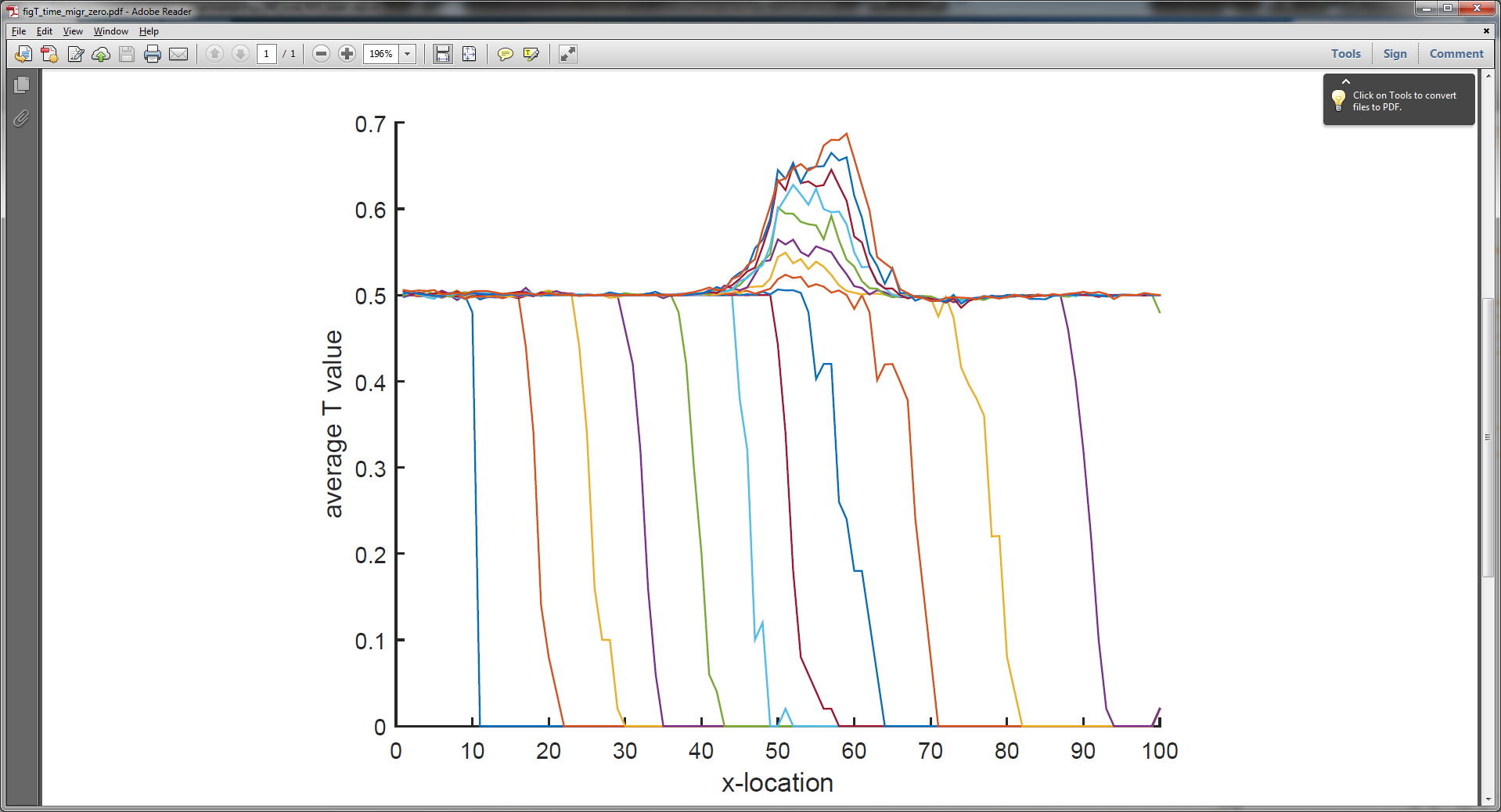


Figure S3. Number of individuals per *x*-location. x are migrants, • are residents, dashed lines indicate the borders of the zone of partial migration, solid line is the weighted mean threshold value *T* per *x*-location. Where this line is missing, population size is zero.


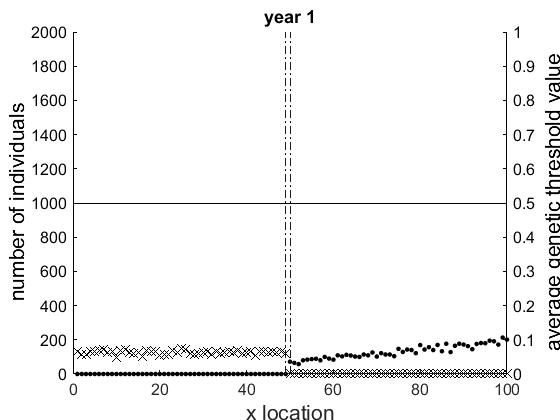

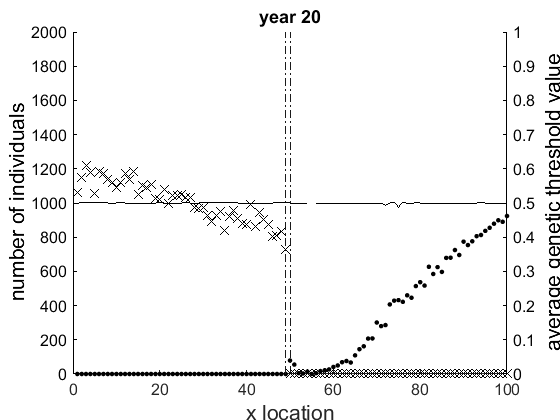


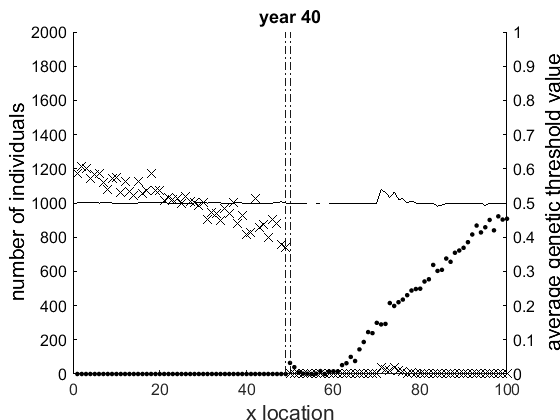

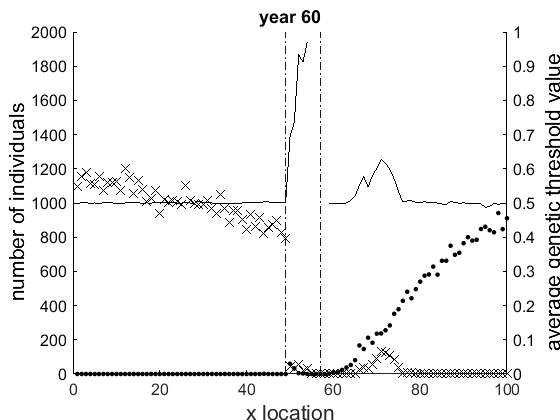


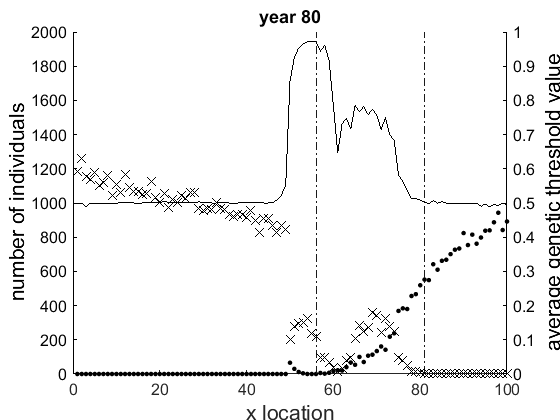

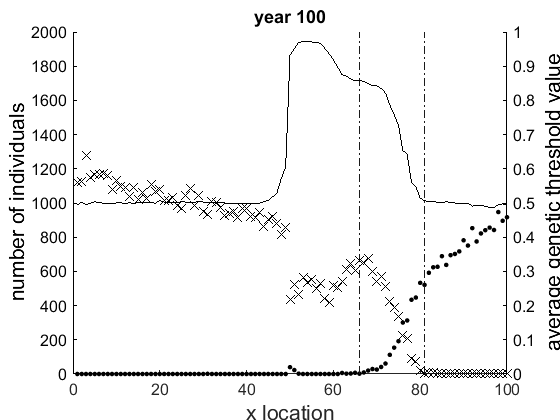


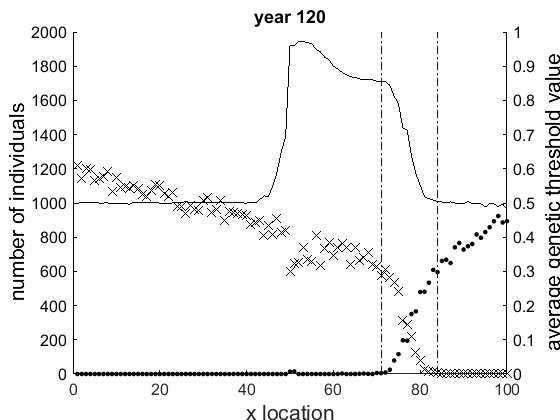

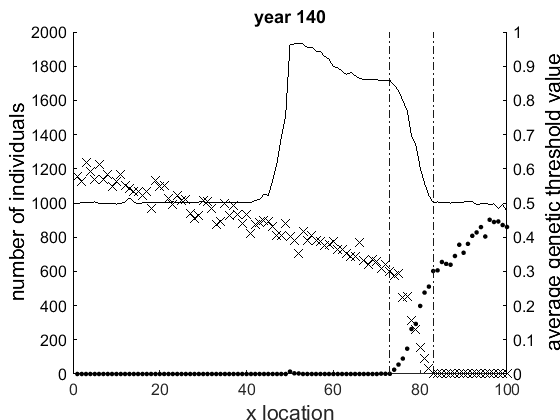


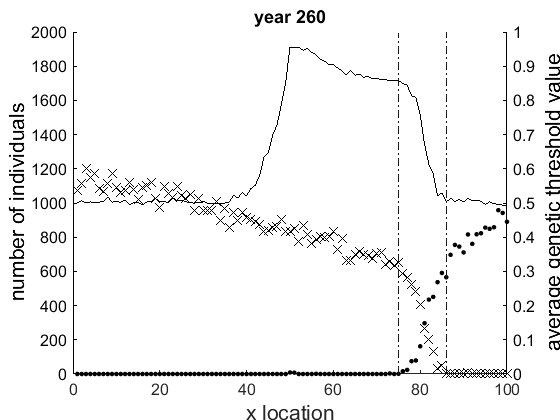

Supplement: Supplementary file 1 [file ECE3-7-8379-s001.docx]
